# Supplementary material for: The genome sequence of the Satin Lutestring moth, Tetheella fluctuosa (Hübner, 1803)
Source: Wellcome Open Res. 2024 Oct 11;9:576. [Version 1] doi: 10.12688/wellcomeopenres.23076.1 (PMC11555359; doi:10.12688/wellcomeopenres.23076.1)
Supplement: Peer review history

# Reviewer report: Reviewer response for version 1

**DOI:** 10.21956/wellcomeopenres.25412.r109226

**Published:** 2024-11-11

**Authors:** Andrew J. Veale

**Affiliations:** 1 Manaaki Whenua Landcare Research, Lincoln, New Zealand

The article provides adequate information on the sample, species, methods and data.  

 There is nothing more required, the genome appears accurate, and the methods used sufficient.

  It appears that the samples were destructively sampled, and no tissue was archived?  Possibly it could be useful to either have a photo of the actual specimen or the location of any archived tissue, but I understand for smaller insects this may not be possible.

Are sufficient details of methods and materials provided to allow replication by others?

Yes

Is the rationale for creating the dataset(s) clearly described?

Yes

Are the datasets clearly presented in a useable and accessible format?

Yes

Are the protocols appropriate and is the work technically sound?

Yes

Reviewer Expertise:

Wildlife genomics

I confirm that I have read this submission and believe that I have an appropriate level of expertise to confirm that it is of an acceptable scientific standard.

# Reviewer report: Reviewer response for version 1

**DOI:** 10.21956/wellcomeopenres.25412.r109213

**Published:** 2024-11-02

**Authors:** Hans-Peter Fuehrer

**Affiliations:** 1 University of Veterinary Medicine, Vienna, Austria

The authors describe the genome sequence of the Satin Lutestring moth, *Tetheella fluctuosa* (Hübner, 1803) within the Darwin Tree of Life project. The manuscript is of relevance. The biology a is described well.

- Please delete globally if this species has a limited distribution.
- Please describe the moth trap used for sampling.

Are sufficient details of methods and materials provided to allow replication by others?

Yes

Is the rationale for creating the dataset(s) clearly described?

Yes

Are the datasets clearly presented in a useable and accessible format?

Yes

Are the protocols appropriate and is the work technically sound?

Yes

Reviewer Expertise:

Medical Entomology/Parasitology

I confirm that I have read this submission and believe that I have an appropriate level of expertise to confirm that it is of an acceptable scientific standard.
